# GSH-C4 Acts as Anti-inflammatory Drug in Different Models of Canonical and Cell Autonomous Inflammation Through NFκB Inhibition

**DOI:** 10.3389/fimmu.2019.00155

**Published:** 2019-02-06

**Authors:** Dolores Limongi, Sara Baldelli, Paola Checconi, Maria Elena Marcocci, Giovanna De Chiara, Alessandra Fraternale, Mauro Magnani, Maria Rosa Ciriolo, Anna Teresa Palamara

**Affiliations:** ^1^Department of Human Sciences and Promotion of the Quality of Life, IRCCS San Raffaele Pisana, San Raffaele Roma Open University, Rome, Italy; ^2^Department of Public Health and Infectious Diseases, Sapienza University of Rome, Rome, Italy; ^3^Institute of Translational Pharmacology, National Research Council Rome, Rome, Italy; ^4^University of Urbino Carlo Bo, Department of Biomolecular Sciences, Urbino, Italy; ^5^Department of Biology, University of Rome “Tor Vergata”, Rome, Italy; ^6^IRCCS San Raffaele Pisana, Rome, Italy; ^7^Institute Pasteur-Fondazione Cenci Bolognetti, Rome, Italy

**Keywords:** glutathione, macrophage, adipocytes, myocytes, cytokine

## Abstract

An imbalance in GSH/GSSG ratio represents a triggering event in pro-inflammatory cytokine production and inflammatory response. However, the molecular mechanism(s) through which GSH regulates macrophage and cell autonomous inflammation remains not deeply understood. Here, we investigated the effects of a derivative of GSH, the N-butanoyl glutathione (GSH-C4), a cell permeable compound, on lipopolisaccharide (LPS)-stimulated murine RAW 264.7 macrophages, and human macrophages. LPS alone induces a significant production of pro-inflammatory cytokines, such as IL-1β, IL-6, and TNF-α and a significant decrement of GSH content. Such events were significantly abrogated by treatment with GSH-C4. Moreover, GSH-C4 was highly efficient in buffering cell autonomous inflammatory status of aged C2C12 myotubes and 3T3-L1 adipocytes by suppressing the production of pro-inflammatory cytokines. We found that inflammation was paralleled by a strong induction of the phosphorylated form of NFκB, which translocates into the nucleus; a process that was also efficiently inhibited by the treatment with GSH-C4. Overall, the evidence suggests that GSH decrement is required for efficient activation of an inflammatory condition and, at the same time, GSH-C4 can be envisaged as a good candidate to abrogate such process, expanding the anti-inflammatory role of this molecule in chronic inflammatory states.

## Introduction

The tripeptide glutathione (GSH) is the most abundant low molecular weight antioxidant in mammalian cells, with a peculiar bond linking the γ*-* carbon of glutamate to the cysteine residue, the thiol group of which is responsible for its function (1). Indeed, intracellularly it is mainly present as a reduced form and two convertible oxidized species: the disulfide form (GSSG) and the mixed disulfide with protein thiols (GSSR). GSH protects cells against exogenous and endogenous harmful molecules including reactive oxygen and nitrogen species (ROS/RNS), limiting the damaging effects of oxidative/nitrosative stress (2, 3). Beside its function as intracellular redox buffer, GSH exerts a key role in the immune system, in antiviral and inflammatory response (4–7). Concerning the inflammatory response, it has been demonstrated that, intracellular GSH depletion represents the first event of the signaling process (8–10). This alteration is accompanied by an increased production of cytokine such as tumor necrosis factor (TNF-α), IL-1β, IL-6, and IL-8 (11, 12). Changes in intracellular GSH levels also characterize the polarization of M1 and M2 macrophages (13). Classical M1 and alternative M2 activation of macrophages, as well as the mirroring Th1-Th2 polarization process of T cells, represents the two extremities of a dynamic changing state characterizing macrophage activation (14).

Cytokines released by M1 macrophages inhibit the proliferation of neighboring cells and promote tissue damage, unlike those derived from M2 macrophages that instead support epithelial cell proliferation and tissue repair. Moreover, microbicidal and tumoricidal activities are intrinsic functions of the M1 macrophages, whereas M2 macrophages are involved in immune tolerance, tissue remodeling, and tumor progression. An imbalance of macrophage M1-M2 polarization is often associated with diseases or inflammatory conditions. Indeed, the M1-M2 switch characterizes the infection by several pathogens, such as bacteria, parasites, and viruses (15). Moreover, several intra-macrophage pathogens switch these cells in M2-type macrophages through the modulation of the intracellular GSH/GSSG ratio. This polarization may provide protection against inflammation and tissue damage; on the other hand, it may skew the immune environment to the advantage of pathogens by supporting their survival. In fact, it was demonstrated that low GSH/GSSG ratio determines altered processing of the antigen, a decrease in IL-12 production and finally a switch from Th1 to Th2 response (16). Contrarily, high GSH/GSSG ratio induced by synthetic molecules in macrophages restores antigen processing and high IL-12 production favoring Th1 response patterns (17). In this context, we recently demonstrated that a GSH derivate (*N*-butanoyl glutathione (GSH) derivative, GSH-C4) was efficient in enhancing the Th1 response toward an antigen, restoring the Th1/Th2 ratio often altered in inflammatory-related processes (18).

These events are mediated by the nuclear factor kappa β transcription factor (NFκB) activation, which regulates the transcription of several pro-inflammatory genes (19). The transcriptional activity of NFκB is finely dependent on GSH levels. Indeed, GSH precursors (e.g., N-acetyl-cysteine, NAC) increased the content of the two NFκB forms (20, 21). Contrarily, other studies show that GSH depletion down-regulates NFκB trans-activation *via* IKK-independent and dependent mechanisms (22).

GSH depletion also represents a key factor in the activation of cell autonomous inflammation, such as in aged-adipose and -skeletal muscle tissues. During aging, visceral adipose tissue (vAT) becomes hypovascularized and resident adipocytes release cytokines and other pro-inflammatory signals, in conjunction with GSH depletion (23–25). Subsequently, secreted chemokines locally attract pro-inflammatory macrophages into the adipose tissue where they form crown-like structures around large dying or dead adipocytes. These tissue macrophages in turn produce cytokines that exacerbate inflammation and degeneration of aged-adipose tissue (26, 27). Similarly, we have recently demonstrated that myoblasts of old mice or cultured differentiated C2C12 myoblasts displayed a decrease of GSH levels accompanied by an increase of pro-inflammatory cytokines such as TNF-α and a decrement of IL-6 (28), which not only regulates myoblast proliferation, but also promotes myoblast differentiation through the p38 MAPK pathway (29). GSH decline could thus impact muscle regeneration efficacy during aging. Thus, GSH/GSSG ratio alteration seems to be a common factor in regulating both macrophages and cell autonomous inflammation.

In the present study, we tested whether by buffering GSH depletion it is possible to counteract the pro-inflammatory response in different cellular models of inflammation. First, we analyzed the effects of GSH-C4 on the inflammatory response induced in LPS-stimulated murine RAW 264.7 macrophages and human primary macrophages. We demonstrated that, GSH-C4 by impeding GSH decrement reduced the expression of pro-inflammatory cytokines *via* NFκB modulation. Subsequent, we analyzed the anti-inflammatory capacity of GSH-C4 in cell autonomous models of inflammation such as aged murine C2C12 myotubes and 3T3-L1 adipocytes, also characterized by a GSH decrement. The results obtained clearly demonstrated an inhibition of NFκB nuclear translocation and cytokine production through inhibition of GSH decrement, suggesting a hypothetical use of GSH-C4 as a drug to attenuate inflammatory responses exerted by cells under different stimuli.

## Materials and Methods

### Cell Culture and Treatments

Murine RAW 264.7 macrophages were acquired from the European Collection of Cell Cultures (Salisbury, UK) and grown in RPMI1640 medium with 10% FBS (Lonza, Basel, CH), 2 mM glutamine, 100 U/ml penicillin/streptomycin and maintained at 37°C in a 5% CO_2_ atmosphere. Cells were plated in 6-well culture plates (1 × 10^6^ cells/well in 3 mL of RPMI with 10% FBS) and incubated at 37°C for 24 hrs. Subsequently, RAW 264.7 macrophages were washed twice with Phosphate Buffered Saline (PBS) (Lonza Sales, Basel, Switzerland) and were treated either with 10 mM GSH-C4 (a kind gift of Redox-Co, Rome, Italy) or 10 mM NAC (Sigma-Aldrich) for 2 hrs. This pre-incubation is used in order to equilibrate the cells with the compound before the challenge with LPS (18). Subsequently, GSH-C4 was removed from culture medium by washing with PBS and the cells were stimulated with 100 ng/ml LPS from E. coli (Sigma Aldrich). After LPS stimulation (1, 3, 6, or 24 hrs) the medium was removed and replaced with fresh medium for further 24 hrs with or without 10 mM GSH-C4 or 10 mM NAC.

Murine 3T3-L1 pre-adipocytes and C2C12 myoblasts were acquired from American Type Cell Culture (ATCC) and grown in DMEM supplemented with 10% newborn serum or FBS, 100 U/ml penicillin/streptomycin, 2 mM glutamine (Lonza Sales, Basel, Switzerland) and maintained at 37°C in a 5% CO_2_ atmosphere. 3T3-L1 and C2C12 cells were plated at density of 2 × 10^5^ cells per well in 6-well plates and differentiated in adipocytes and myotubes, respectively, as previously reported (28, 30).

### Isolation of Nuclei

Cell pellets were lysed in nucleus lysis buffer (NLB) containing 50 mM Tris-HCl pH 8.1, 10 mM EDTA, 1% SDS, 10 mM sodium butyrate, protease inhibitors, and incubated 1 hrs at 4°C. After centrifugation at 600 x g for 5 min at 4°C the nuclear pellets were resuspended in 1 ml of NLB. Subsequently, nuclei were purified on NLB containing 30% sucrose (w/v) and centrifuged at 700 x g for 10 min (31, 32). The purified nuclei were resuspended in NLB to eliminate nuclear debris and finally used for Western blot or ChIP assays.

### Western Blot Analysis

Cell pellets were lysed in RIPA buffer (50 mM Tris-HCl, pH 8.0, 150 mM NaCl, 12 mM deoxycholic acid, 0.5% Nonidet P-40, and protease inhibitors). Protein samples were used for SDS-PAGE followed by Western blotting as previously described (28). Nitrocellulose membranes were stained with primary antibodies against Tubulin (1:1,000), p-NFκB (p65) (Ser536) (1:1,000), NFκB (p65) (1:1,000), NFκB (p50) (1:1,000); IKB-α (1:500), IKK-α/β (1:500), p-IKB-α (1:500), p-IKK-α/β (1:500) LDH (1:1,000), Sp1 (1:500), TNF-α (1:500), p-p38 (1:1,000), p-ERK1/2 (1:1,000) (Santa Cruz Biotechnology). The nitrocellulose membranes were incubated with the appropriate horseradish peroxidase conjugated secondary antibody (Bio-Rad), and immunoreactive bands were detected by a Fluorchem Imaging System upon staining with ECL Select Western Blotting Detection Reagent (GE Healthcare, Pittsburgh, PA, USA; RPN2235). The Western blots reported are from one experiment out of three separated experiments that gave similar results.

Proteins were assayed by the method described by Lowry et al. (33).

### RT-qPCR Analysis

TRI Reagent (Sigma-Aldrich) was used to extract total RNA, which was used for retro-transcription. qPCR was performed in triplicate by using validated qPCR primers (BLAST), Ex TAq qPCR Premix (Lonza Sales) and the Roche Real Time PCR LightCycler II (Roche Applied Science, Monza, Italy). mRNA levels were normalized to ribosomal protein large subunit (RPL) and the relative mRNA levels were determined by using the 2^−ΔΔ*Ct*^ method (34). The primer sequences are listed in Table 1.

**Table 1:** List of primers used for RT-qPCR and ChIP analysis.

**Genes**	**Sequences**
IL-1β FW	5′-GCTGAAAGCTCTCCACCTCA−3′
IL-1β RV	5′- GCTTGGGATCCACACTCTCC-3′
IL-6 FW	5′-CTCTGCAAGAGACTTCCATCCA−3′
IL-6 RV	5′-GACAGGTCTGTTGGGAGTGG−3′
TNF-α FW	5′-GCCTCTTCTCATTCCTGCTTG−3′
TNF-α RV	5′- CTGATGAGGGAGGCCATT-3′
NFκB FW	5′- GAAATTCCCTGATCCAGACAAAAAC-3′
NFκB RV	5′-ATCACTTCAATGGCCTCTGTGTAG−3′
IL-10 FW	5′-ATAAACTGCACCCCACTTCCCA-3′
IL-10 RV	5′-TGGACCATCTTCACTACGGG-3′
MCP-1 FW	5′-GCTCAGCCAGATGCAGTTAA-3′
MCP-1 RV	5′-TCAAAACAGTGGTTCGAGTTCT-3′
β-Actin FW	5′-CACACCCGCCACCAGTTCGC-3′
β-Actin RV	5′-TTGCACATGCCGGAGCCGTT−3′
NFκB FW (ChIP) FW	5′-GTCGAGTATGGGGACCC-3′
NFκB RV (ChIP) RV	5′-GGAATGGGTTACAGG-3′

### Chromatin Immunoprecipitation Assay

ChIP was carried out according to the protocol of Im et al. (35) with some modifications. After crosslinking, the nuclei extracted from RAW 264.7 macrophages were fragmented by ultrasonication using 4 × 15 pulse (output 10%, duty 30%). Samples were precleared with pre-adsorbed salmon sperm Protein G agarose beads (1 hrs, 4°C). Subsequently, the samples were subjected to overnight immunoprecipitation using anti-NFκB antibody. After de-cross-linking (1% SDS at 65°C for 3 hrs), qPCR was used to quantify the promoter binding with 30 cycles total (95°C, 1 s; 60°C, 30 s; 72°C, 60 s). Results are expressed as percentage of Input values (1%). The primers used are reported in Table 1.

### Measurement of Cytokine Production

The supernatants were removed at the allotted times and the level of IL-1β, IL-6, and TNF-α production was quantified using Luminex Assay (Bio-Rad) and Elisa Kit (ENZO LifeScience) according to the manufacturer's instructions (36, 37).

### Determination of GSH and GSH-C4

Intracellular GSH was assayed upon formation of S-carboxymethyl derivatives of the free thiol with iodoacetic acid, followed by the conversion of free amino groups to 2,4-dinitrophenyl derivatives by the reaction with 1-fluoro-2,4-dinitrobenzene and quantified through high performance liquid chromatography (HPLC) as previously described (28). RAW 264.7 macrophages were treated with 100 ng/ml LPS for the indicated time and immediately used for GSH determination. Intracellular GSH-C4 was determined as previously described (18). Data are expressed as nmol of GSH/mg protein.

### Analysis of Cell Viability and Proliferation

Adherent cells were detached with trypsin, washed with PBS and directly counted by optical microscope on hemocytometer, after Trypan Blue staining.

### Human Macrophages Culture and Differentiation

The peripheral blood mononuclear cells (PBMCs) from healthy controls were separated by density gradient according to Lympholyte® Cell Separation Media (Cedarlane) technique. PBMCs were immediately re-suspended in RPMI 1640 medium supplemented with 10% FBS (Lonza, Basel, CH), 2 mM glutamine, 100 U/ml penicillin/streptomycin in the presence of 50 ng/ml human recombinant granulocyte macrophage colony-stimulating factor (38), seeded in 24-well plates (4 × 10^6^ cells/well; 1 ml), and maintained at 37°C in a 5% CO_2_ atmosphere for up to 2 weeks. Cell culture media was replenished every 3 days and cells monitored morphologically for differentiation. Geimsa staining of macrophages was accomplished to visualize the typical morphology under light microscopy.

### Fluorescence Microscopy

RAW 264.7 macrophages grown on glass coverslips were fixed with 4% paraformaldehyde and permeabilized with 0.4% Triton X-100. Cells were incubated with a monoclonal anti-p-NFκB (p65) (1:50) diluted in PBS containing 10% FCS and then probed with the appropriate Alexa Fluor®-conjugated secondary antibody. Nuclei were stained with the vital dye Hoechst 33342. Images of cells were digitized with a Cool Snap video camera connected to Nikon Eclipse TE200 epifluorescence microscopy (Nikon, Firenze, Italy). All of images were captured under constant exposure time, gain and offset.

### Statistical Analyses

The results are presented as means ± S.D. Statistical evaluation was conducted by ANOVA, followed by the post-Student-Newman-Keuls. Differences were considered to be significant at *p* ≤ 0.05. *N* = 3 or other numbers is referred to the number of independent experiment performed. Each experiments referring to cytokines determination or other mRNA levels detection was done in triplicate.

## Results

### Effects of LPS on Cytokine Production and GSH Level in RAW 264.7 Macrophages

**Figure 1:**
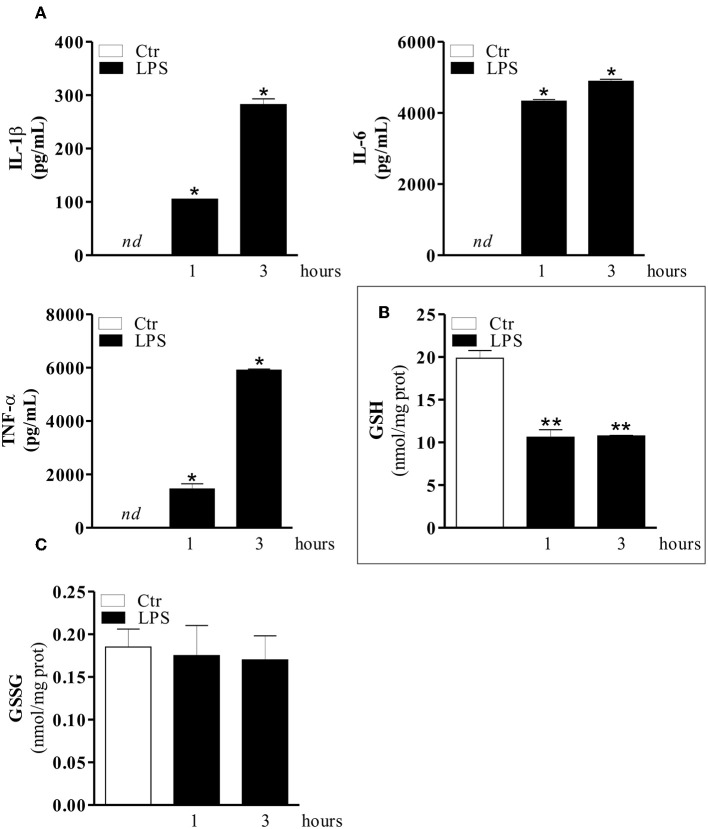
LPS induces an increase of pro-inflammatory cytokines and a concomitant decrease of GSH level. **(A)** RAW 264.7 macrophages were treated with 100 ng/ml LPS for 1 and 3 hrs. After 1 and 3 hrs the medium with LPS was replaced with fresh medium for 24 hrs. IL-1β, IL-6, and TNF-α production in culture supernatants were detected using Luminex Assay (Bio-Rad). Data are expressed as means ± S.D. (*n* = 4; *p < 0.01). **(B)** RAW 264.7 macrophages were treated with 100 ng/ml LPS for 1 and 3 hrs. Immediately, after 1 and 3 hrs of LPS treatment, GSH content was assayed by HPLC. Data are expressed as means ± S.D. (*n* = 3; **p < 0.001). **(C)** RAW 264.7 macrophages were treated with 100 ng/ml LPS for 1 and 3 hrs. Immediately, after 1 and 3 hrs of LPS treatment GSSG content was assayed by HPLC. Data are expressed as means ± S.D. (*n* = 3). *nd*, not determined. All the images reported in the figures are representative of at least three experiments that gave similar results.

It is well-known that the GSH-redox equilibrium is fundamental for mounting efficient innate immune response in macrophages and that LPS is a strong macrophage activator, which stimulates the secretion of various cytokines (39, 40). Therefore, in order to determine whether modulation of GSH content during inflammation could be used as a therapeutic approach we firstly determined the cytokine production and GSH levels in RAW 264.7 macrophages treated with LPS. Figure 1A shows the levels of IL-1β, IL-6, and TNF-α at different time-points of LPS treatment, by the Luminex Assay. The obtained data outlined a significant production of cytokines already after 1 hrs LPS treatment with peaked values around the 3 or 6 hrs (Supplemental Figure 1A) compared to control cells. At longer incubation times (i.e., 24 hrs), cytokine production decreased and cell viability was seriously compromised, as reported in Supplemental Figures 1A,B. We also measured the intracellular GSH levels after stimulation with LPS for 1, 3, and 6 hrs by HPLC. Figure 1B shows a decrement of GSH at both 1 and 3 hrs of LPS treatment compared to control cells, whereas no changes in the oxidation form of GSH (GSSG) were observed (Figure 1C). The same trend was observed at 6 hrs (Supplemental Figure 1C). Based on this result, we used 1 hrs LPS-stimulation for the subsequent experiments. Thus, intracellular GSH rather than GSSG content is mainly affected during macrophage activation and cytokine production, supporting the idea that inflammatory response might be modulated by buffering GSH decrement.

### Effects of GSH-C4 on LPS-Induced Cytokine Production and GSH Modulation

**Figure 2:**
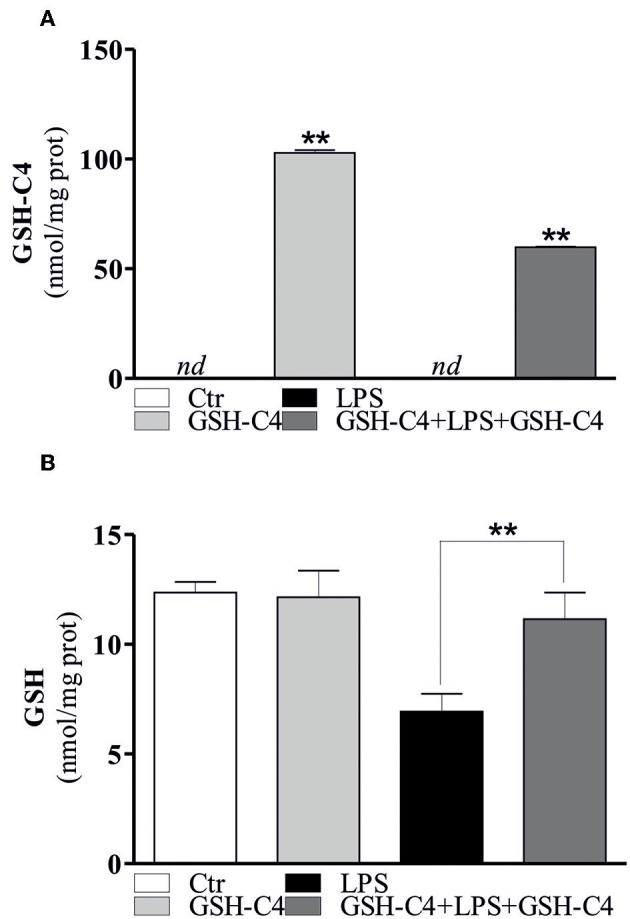
GSH-C4 is able to reverse the increase of intracellular GSH levels after LPS stimulation. **(A)** RAW 264.7 macrophages were treated with 10 mM GSH-C4 for 2 hrs. Subsequently, GSH-C4 was removed from culture medium and the cells were stimulated with 100 ng/ml LPS for 1 hrs. After 1 hrs RAW 264.7 macrophages were treated again with 10 mM GSH-C4 or with fresh medium for 24 hrs. GSH-C4 content was assayed by HPLC. Data are expressed as means ± S.D. (*n* = 3; **p < 0.001). **(B)** GSH content was assayed by HPLC. Data are expressed as means ± S.D. (*n* = 4; **p < 0.001). *nd*, not determined. All the images reported in the figures are representative of at least three experiments that gave similar results.

**Figure 3:**
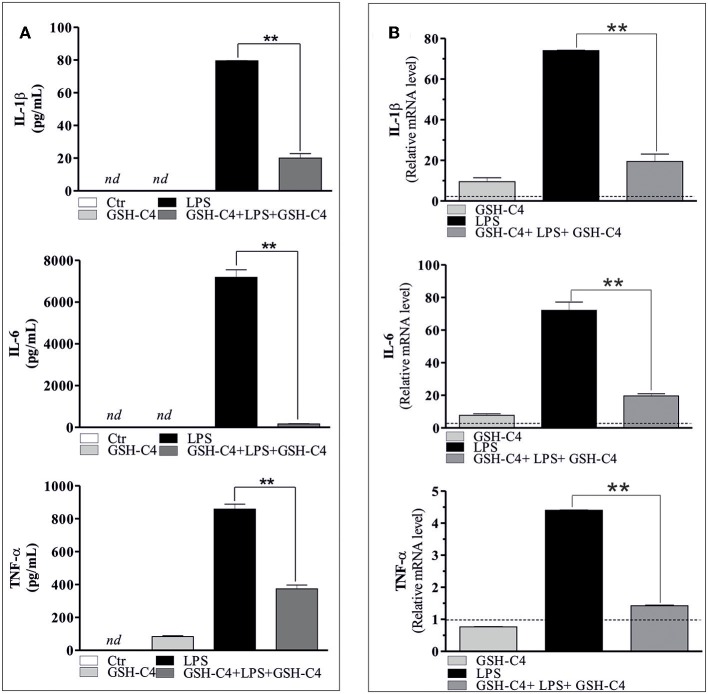
GSH-C4 treatment inhibits the production and expression of pro-inflammatory cytokines after LPS stimulation in RAW 264.7 macrophages. **(A)** RAW 264.7 macrophages were treated with 10 mM GSH-C4 for 2 hrs. Subsequently, GSH-C4 was removed from culture medium and the cells were stimulated with 100 ng/ml LPS for 1 hrs. After 1 hrs RAW 264.7 macrophages were treated again with 10 mM GSH-C4 or with fresh medium for 24 hrs. IL-1β, IL-6, and TNF-α production in culture supernatants were detected using Luminex Assay (Bio-Rad). Data are expressed as means ± S.D. (*n* = 3; **p < 0.001). **(B)** Total RNA was isolated and relative mRNA levels of IL-1β, IL-6, and TNF-α were analyzed by RT-qPCR. mRNA levels were normalized to ribosomal protein large subunit (RPL). Dashed line indicates the value of control. Data are expressed as means ± S.D. (*n* = 6; **p < 0.001). *nd*, not determined. All the images reported in the figures are representative of at least three experiments that gave similar results.

Taking advantage of the possibility to use a cell permeable GSH derivative (GSH-C4) to increase intracellular GSH levels, we treated RAW 264.7 cells with GSH-C4 and evaluated both inflammatory response and cytokine levels. In particular, we pre-treated the cells with GSH-C4 (10 mM) for 2 hrs. Subsequently, GSH-C4 was removed from culture medium and the cells were treated with 100 ng/ml LPS for 1 hrs. After stimulation, RAW 264.7 cells were treated again with 10 mM GSH-C4 for 24 hrs. The levels of intracellular thiols and cytokine production were determined. As expected, intracellular GSH-C4 was determined only in GSH-C4 treated cells and in cells co-treated with LPS (Figure 2A). Moreover, the treatment with GSH-C4 was able to buffer the intracellular GSH depletion observed in RAW 264.7 macrophages upon LPS stimulation (Figure 2B). Subsequently, we measured the concentration of inflammatory cytokines in the culture supernatants with the Luminex Assay. IL-1β, IL-6, and TNF-α production was decreased by GSH-C4 with respect to LPS stimulation (Figure 3A). This result was confirmed by the analysis of intracellular mRNA (Figure 3B). To assess the efficacy of GSH-C4, we carried out the same set of experiments with N-acetyl-L-cysteine (NAC), a recognized pro-GSH synthesis molecule. As shown in Supplemental Figure 1D, NAC treatment (10 mM) was able to affect cytokine production but at lower extent with respect to GSH-C4.

### Effects of GSH-C4 on LPS-Mediated Nuclear Translocation

**Figure 4:**
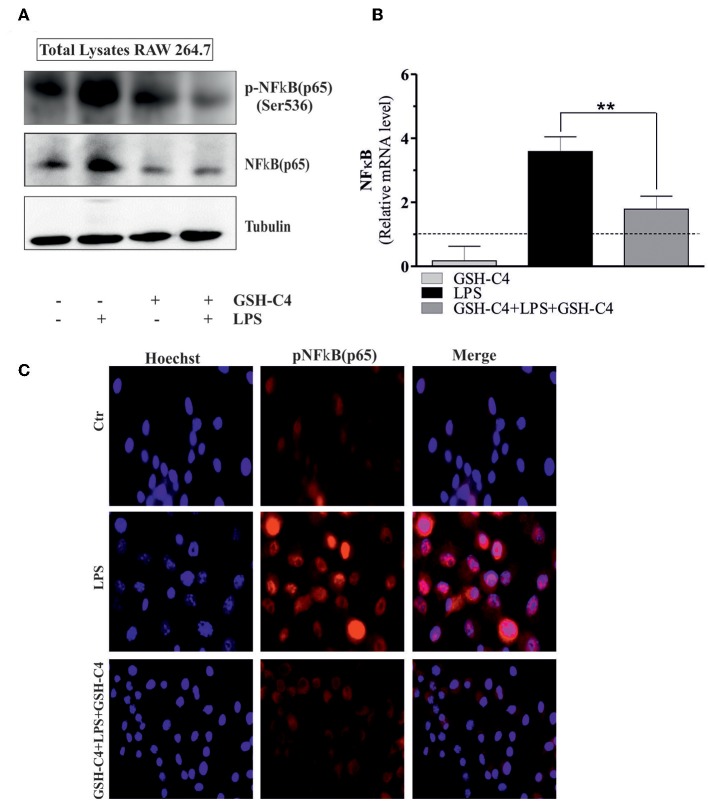
GSH-C4 treatment reduces the NFκB (p65) induction in LPS-stimulated RAW 264.7 macrophages. **(A)** RAW 264.7 macrophages were treated with 10 mM GSH-C4 for 2 hrs. Subsequently, GSH-C4 was removed from culture medium and the cells were stimulated with 100 ng/ml LPS for 1 hrs. After 1 hrs RAW 264.7 macrophages were treated again with 10 mM GSH-C4 or fresh medium for 24 hrs. Twenty micrograms of total proteins were loaded for Western blot analysis of the phosphorylated [p-NFκB (p65)] and total form of NFκB [NFκB (p65)]. Tubulin was used as loading control. **(B)** Total RNA was isolated and relative mRNA level of NFκB was analyzed by RT-qPCR. Dashed line indicates the value of control. mRNA levels were normalized to RPL. Data are expressed as means ± S.D. (*n* = 4; **p < 0.001). **(C)** Paraformaldeyde-fixed cells were subjected to immunostaining with anti-p-NFκB (p65) antibody (red, AlexaFluor568®). Nuclei were stained with Hoechst 33342 (blue). Merge represents the overlay of nuclei and p-NFκB (p65) staining. Images reported are from one experiment representative of three that gave similar results. All the immunoblots reported are from one experiment representative of three that gave similar results.

**Figure 5:**
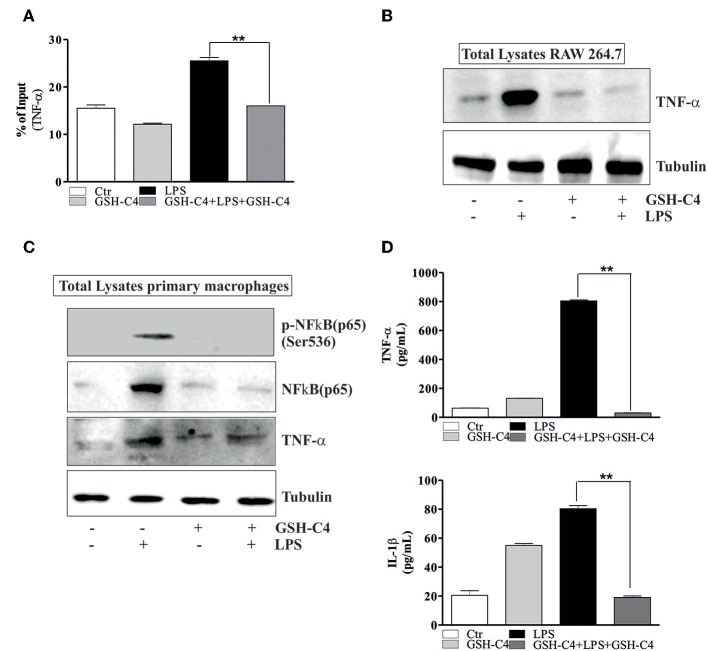
GSH-C4 treatment prevents the transcription of TNF-α. **(A)** RAW 264.7 macrophages were treated with 10 mM GSH-C4 for 2 hrs. Subsequently, GSH-C4 was removed from culture medium and the cells were stimulated with 100 ng/ml LPS for 1 hrs. After 1 hrs RAW 264.7 cells were treated again with 10 mM GSH-C4 or fresh medium for 24 hrs. ChIP assay was carried out on crosslinked nuclei using NFκB antibody followed by qPCR analysis of NFκB binding site on TNF-α promoter (+382: ggaggagaTTCCttg). Data are expressed as means ± SD (*n* = 3; **p < 0.001). **(B)** Twenty micrograms of total proteins were loaded for Western blot analysis of the TNF-α. Tubulin was used as loading control. **(C)** 4 × 10^6^ cells/well human macrophages were treated with 10 mM GSH-C4 for 2 hrs. Subsequently, GSH-C4 was removed from culture medium and the cells were stimulated with 100 ng/ml LPS for 1 hrs. After 1 hrs human macrophages were treated again with 10 mM GSH-C4 or fresh medium for 24 hrs. Twenty micrograms of total proteins were loaded for Western blot analysis of the phosphorylated and total form of NFκB [p-NFκB (p65), NFκB (p65)] and TNF-α. Tubulin was used as loading control. **(D)** TNF-α and IL-1β production in culture supernatants were detected using Elisa Kit (ENZO LifeScience). Data are expressed as means ± S.D. (*n* = 3; **p < 0.001). All the immunoblots reported are from one experiment representative of three that gave similar results.

NFκB-mediated pathways are implicated in the activation of LPS-induced inflammatory mediators and cytokine production (18, 41). Therefore, we investigated whether NFκB was affected by treatment with GSH-C4. Relative to controls, LPS stimulation significantly increased the phosphorylation of NFkB [p-NFkB (p65) Ser536] and levels of total NFkB after 1 hrs stimulation (Figure 4A). The treatment with GSH-C4 efficiently abrogated these changes in NFκB activation/accumulation (Figure 4A). Consistently, using RT-qPCR, we detected a corresponding increase in the transcriptional level of NFkB upon LPS stimulation, also inhibited by GSH-C4 (Figure 4B). To assess whether other signaling pathways are activated by LPS treatment we measured the levels of members of MAPKs pathways, such as p38 and ERK1/2 due to their involvement in inflammation (42, 43). RAW 264.7 cells were treated with 100 ng/ml LPS for 1 and 3 hrs and immediately used for Western blot analysis of the basal and phosphorylated form of p38 and ERK1/2. No significant changes in the activation and levels of these kinases were detected relative to untreated controls (Supplemental Figure 2A), confirming the main role played by NFκB in our experimental system. To further evaluate the involvement of NFκB signaling pathway in LPS-stimulated macrophages we determined its nuclear localization by Immunofluorescence microscopy. Phosphorylated NFkB (p65) mainly localized in the nucleus after LPS stimulation compared to unstimulated cells, a process efficiently inhibited by GSH-C4 treatment (Figure 4C). These data were also confirmed by Western blot analysis of both p-NFκB (p65) and total NFκB on purified nuclei and cytoplasmatic fractions from LPS- and GSH-C4-treated RAW 264.7 macrophages (Supplemental Figure 2B). Interestingly, the cytoplasmic levels of p-NFkB (p65) remained high and largely unaffected in the presence of GSH-C4, independent of LPS stimulation. To further investigate whether the decreased levels of NFκB were directly related to the decrement in cytokine release, we analyzed the NFκB-mediated TNF-α production. In fact, some studies in literature reported a close transcriptional correlation between NFκB and TNF-α (44). For this reason, we analyzed the mouse TNF-α promoter using Genomatix Software Suite database to identify NFκB consensus sequences (known as κB sites). We found five NFκB consensus sequences in the TNF-α promoter, located at −83, +382, −400, +624, and −805. A ChIP analysis of these sequences was performed to clarify the regulatory role of NFkB on the murine TNF-α promoter. This demonstrated a significant and selective increase in NFkB occupancy on the +382 region of the TNF-α promoter after LPS stimulation, compared to unstimulated controls (Figure 5A), while −83, −400, +624, and −805 regions did not show NFκB binding (data not shown). Accordingly, we observed that after GSH-C4 treatment the occupancy of NFκB on its consensus sequence located on TNF-α gene promoter was significantly decreased with respect to LPS-stimulated macrophages (Figure 5A). This transcriptional regulation affected the mRNA and protein levels of TNF-α, both significantly decreased following treatment with GSH-C4 (Figures 3B, 5B). Finally, in order to assess the specificity of NFκB in the LPS-mediated pro-inflammatory response we determined the expression level of an anti-inflammatory cytokine, IL-10 the transcription of which is also under NFκB (45). The mRNA levels of IL-10 significantly increased after LPS stimulation, a process efficiently inhibited upon GSH-C4 treatment (Supplemental Figure 2C). Moreover, GSH-C4 treatment efficiently abrogates the IL-10 transcriptional increment. To test the validity of our results on primary cells, we examined the effects of GSH-C4 on an *ex vivo* experimental model represented by primary macrophage derived from PBMCs. After 2 weeks of differentiation, primary macrophages were pre-treated with GSH-C4 (10 mM) for 2 hrs. Thereafter, GSH-C4 was removed from culture medium and the macrophages treated with 100 ng/ml LPS for 1 hrs. After stimulation, cells were supplemented with medium containing 10 mM GSH-C4 for 24 hrs. LPS induced a significant increase of the phospho-active form of NFkB (p-NFkB (p65) Ser536) as well as of the basal form (NFκB) relative to unstimulated cells (Figure 5C). As seen in the case of RAW 264.7 cells, treatment with GSH-C4 efficiently abrogated these changes. As a downstream effect, we detected an increase in the protein levels of TNF-α, also reversed to basal levels upon GSH-C4 treatment (Figure 5C). Finally, we analyzed the levels of principal pro-inflammatory cytokines and, as shown in Figure 5D, the increased levels of intracellular TNF-α and IL-1β were completely abolished upon GSH-C4 treatment with respect to LPS-stimulated cells, suggesting a broad effect of this compound in counteracting NFκB activation.

### GSH-C4 Reverts Activation and Cytokine Production in Cell Autonomous Induced Inflammation

**Figure 6:**
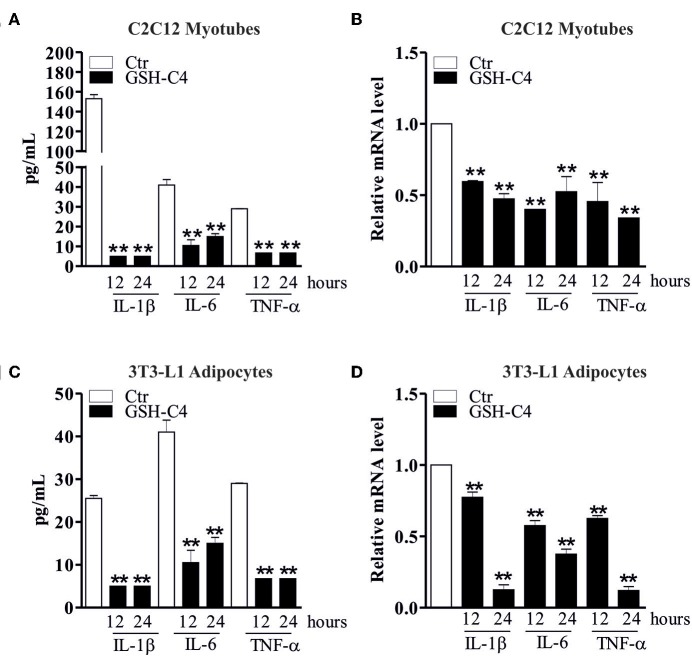
GSH-C4 treatment abrogates the production and expression of pro-inflammatory cytokines in C2C12 myotubes and 3T3-L1 adipocytes. **(A)** C2C12 cells were differentiated for 4 days. Subsequently, 10 mM GSH-C4 was added to the myotubes for 12 or 24 hrs. IL-1β, IL-6, and TNF-α production in culture supernatants were detected using Luminex Assay (Bio-Rad). Data are expressed as means ± S.D. (*n* = 4; **p < 0.001). **(B)** Total RNA was isolated and relative mRNA levels of IL-1β, IL-6, and TNF-α were analyzed by RT-qPCR. mRNA levels were normalized to RPL. Data are expressed as means ± S.D. (*n* = 6; **p < 0.001). **(C)** 3T3-L1 cells were differentiated for 8 days. Subsequently, 10 mM GSH-C4 was added to the adipocytes for 12 or 24 hrs. IL-1β, IL-6, and TNF-α production in culture supernatants were detected using Luminex Assay (Bio-Rad). Data are expressed as means ± S.D. (*n* = 3; **p < 0.001). **(D)** Total RNA was isolated and relative mRNA levels of IL-1β, IL-6, and TNF-α were analyzed by RT-qPCR. mRNA levels were normalized to RPL. Data are expressed as means ± S.D. (*n* = 4; **p < 0.001). All the images reported in the figures are representative of at least three experiments that gave similar results.

**Figure 7:**
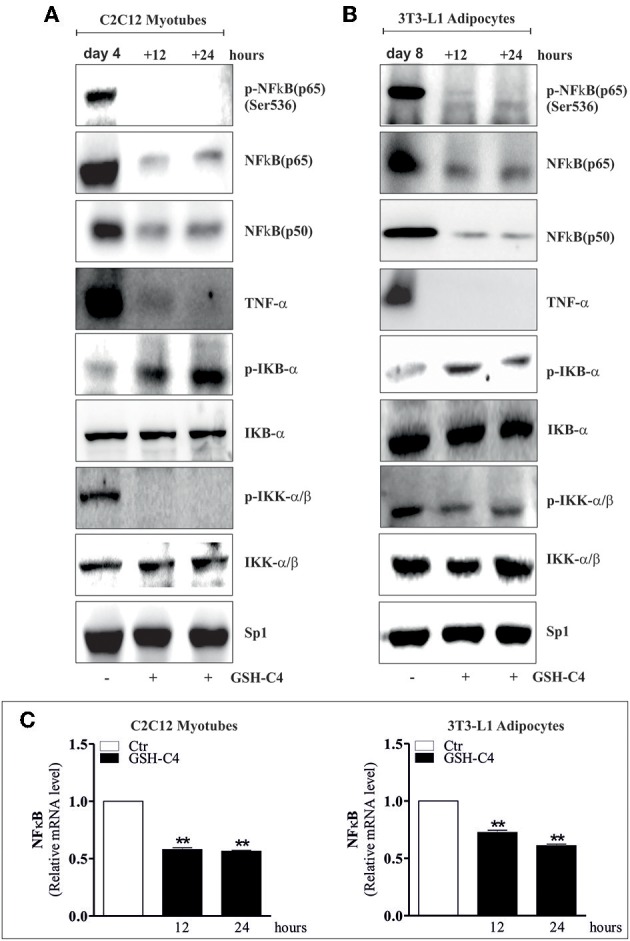
GSH-C4 treatment prevents the induction of NFκB-signaling pathway in C2C12 myotubes and 3T3-L1 adipocytes. **(A,B)** C2C12 and 3T3-L1 cells were differentiated for 4 and 8 days, respectively. Subsequently, 10 mM GSH-C4 was added to the cells for 12 or 24 hrs. Twenty micrograms of total proteins were loaded for Western blot analysis of the phosphorylated and total form of NFκB [p-NFκB (p65), NFκB (p65)], NFκB (p50), TNF-α, p-IKB-α, p-IKK-α/β, IKB-α, and IKK-α/β. Sp1 was used as loading control. **(C)** Total RNA was isolated and relative mRNA levels of NFκB were analyzed by RT-qPCR. mRNA levels were normalized to RPL. Data are expressed as means ± S.D. (*n* = 4; **p < 0.001). All the immunoblots reported are from one experiment representative of three that gave similar results.

Many diseases and disorders, such as type 2 diabetes, atherosclerosis, obesity, sarcopenia, and myopathies myopathies are accompanied by inflammation or increase of the inflammatory cytokines (25, 28, 46–48). In this context, we have previously demonstrated a significant IL-6 and TNF-α increase in visceral adipose tissue of 24 month-old mice and *in vitro* aged adipocytes (25). Moreover, we have also shown the same activation in skeletal muscle of old mice (28). Thus, we evaluated whether GSH-C4 also affects cell autonomous inflammation-associated processes in these experimental systems. In particular, 3T3-L1 murine pre-adipocytes were differentiated for 8 days and then treated with 10 mM GSH-C4 for 12 and 24 hrs. C2C12 murine myoblasts were differentiated for 4 days and subsequently treated with 10 mM GSH-C4 for 12 and 24 hrs. Similar to our above results with macrophages, treatment with GSH-C4 efficiently inhibited the production of cytokines (IL-1b, IL-6, and TNF-α), compared to undifferentiated 3T3-L1 and C2C12 cells, both at 12 and 24 hrs (Figures 6A,C). Similar significant effects were also detected at the cytokine mRNA levels (Figures 6B,D). These results confirmed the anti-inflammatory capacity of GSH-C4 also in cell autonomous mediated inflammation. We then investigated whether the NFkB signaling pathway detected in macrophages, was also induced in cell autonomous systems, by measuring protein levels of inhibitory/activator intracellular signaling mediators. For this reason, we analyzed the protein levels of inhibitory/activator partners. In particular, we evaluated the transcriptional phosphoactive form of NFκB and its inhibitory partner IKB-α, which sequestrates NFκB in the cytoplasm (49). Moreover, we analyzed the IκB kinase (IKKα/β), which by phosphorylating IKB-α leads to its degradation (50). GSH-C4 decreased levels of p-NFkB (p65), NFkB (p65), NFkB (p50) in myotubes, and adipocytes relative to baseline levels detected in control cells (Figure 7C). Interestingly, this was paralleled by increased inhibitory protein p-IKB-α and decreased p-IKK-α/β (Figures 7A,B).

The potential anti-inflammatory role of GSH-C4 was additionally tested on MCP-1, a chemokine autonomously released by adipocytes. Indeed, it has been demonstrated that an increase of MCP-1 in adipose tissue determines a greater infiltration of macrophages into the tissue (51). Supplemental Figure 2D demonstrates that during adipocytes differentiation MCP-1 mRNA levels were easily detectable and that GSH-C4 treatment resulted in a significant reduction of those levels with respect to control cells, confirming its anti-inflammatory role also in adipose tissue-related inflammation charactering obesity (52).

## Discussion

A strong connection exists between accumulation of free radicals (ROS/RNS) and the modifications of the immune response and inflammation (53). In particular, GSH depletion is envisaged as one of the first events of inflammatory response activation (8, 10, 54). Therefore, we evaluated the outcome of restraining intracellular GSH homeostasis in different inflammatory conditions, as a potential anti-inflammatory strategy. Indeed, the use of GSH precursor or antioxidants was previously reported to restore cytokine synthesis and the activation of inflammatory pathways. In particular, treatment with NAC reestablished GSH levels and pro-inflammatory cytokines production in different experimental systems (55, 56). Contrarily, treatment with buthionine sulfoximine (BSO), an inhibitor of γ-glutamyl-l-cysteinyl-ethyl ester (γ-GCS), the rate-limiting enzyme in the biosynthesis of GSH, has the potential to enhance cytokine secretion by up-regulating ROS level (57). So, it is clear that the maintenance of GSH homeostasis may represent a therapeutic treatment in many diseases where oxidative/nitrosative stress and thiols alterations play a key role in the pathophysiology.

In the current study, in line with other works demonstrating a significant decrease of GSH levels in LPS-treated mice (54, 58–61), we demonstrated that GSH-redox balance and pro-inflammatory cytokines production were affected in LPS stimulated macrophages.

GSH-C4, a permeable GSH derivative, previously used as an antiviral and immune-modulator in different models (62–65), efficiently counteracted the LPS-mediated inflammatory response. Preliminary experiments fixed at 10 mM the non-toxic concentration of GSH-C4 able to restore different levels of GSH depletion, induced by various GSH-depleting agents, ranging from moderate depletion (about 50%) to severe depletion (about 80%). Indeed, the results obtained indicate that 10 mM GSH-C4, as such, reached 30% of the intracellular thiols content, confirming its ability to easily enter the cell; moreover, GSH-C4 was able to restore the LPS-depleted intracellular GSH levels. We also showed that GSH-C4 addition significantly inhibited LPS-induced IL-1β, IL-6, and TNF-α production. GSH-C4 anti-inflammatory capacity was higher than that exerted by the antioxidant NAC as demonstrated by the very efficient inhibition of pro-inflammatory cytokine production, confirming its strong anti-inflammatory action. We speculate that the stronger anti-inflammatory activity observed in GSH-C4-treated cells is due to the thiol species supplemented. More exactly, both NAC (data not shown) and GSH-C4 can restore GSH content in GSH-depleted cells; the excess of thiol species measured in NAC-treated and GSH-C4-treated cells were NAC and GSH-C4 respectively. Thus, we propose that the action exerted by GSH-C4 may be ascribed to the GSH-C4 itself. However, further studies are required to address the exact mechanism.

It is well-established that the canonical inflammatory signaling transduction pathway includes NFκB activation (66). NFκB induces cytokine production that modulate the immune response (such as TNF-α, IL-1, IL-6, and IL-8) as well as the production of adhesion molecules, which drive the recruitment of leukocytes to the inflammation sites (67). NFκB resides in the cytoplasm as an inactive heterodimeric form of two subunits (p50 and p65). In particular, NFκB heterodimer is sheltered by an inhibitory subunit, IKB-α that prevents its nuclear translocation. Under specific stimulation, IKB-α can be phosphorylated, ubiquitinated, and degraded via the proteasome, thus releasing NFkB, which can then translocate to the nucleus to drive transcription of genes involved in the inflammatory response (66, 68). Our results confirmed that, upon LPS treatment, p-NFkB (p65) translocates to the nucleus to support the inflammatory response. In this context, GSH-C4 inhibits the expression and release of IL-1β, IL-6, and TNF-α through blocking p-NFkB (p65) activation. Indeed, we showed that GSH-C4 acts as a transcriptional inhibitor of NFkB (p65), further interfering with its nuclear localization. In our experiments, the involvement of NFkB appeared preponderant over that of other intracellular signaling mediators involved in inflammation. For instance, as MAPKs play a central role in signal transduction pathways during inflammation (42, 43), we tested the possible activation of p38 and ERK1/2 in LPS-mediated inflammatory response. However, no significant changes in the phosphorylated active forms of these kinases were detected, excluding their involvement in this process.

TNF-α increased expression is a hallmark in numerous inflammatory diseases and also in the inflammatory response to LPS (69–71). NFκB had a direct role in the stimulation of TNF-α gene transcription, demonstrated by the presence of NFκB binding motifs in the TNF-α promoter, which are recognized by the transcription factor in response to different stimuli (44, 72). In the present work we demonstrated the ability of p-NFκB (p65) to bind the +382 RE on the murine TNF-α promoter allowing increased TNF-α expression. Our results indicated that GSH-C4 can reverse this process thus inhibiting inflammation. Indeed, it was previously reported that specific residues of cysteine of NFkB are implicated in recognition of specific DNA regions and that redox-mediated mechanisms have regulatory role in the NFκB-mediated gene expression (73, 74). In fact, we previously found that 2 hrs-pre-treatment with GSH-C4 regulated NFκB DNA binding activity favoring and prolonging its association with IL-12 p40 promoter sequence (18). In this paper, the prolonged GSH-C4 treatment for 24 hrs may create an altered oxidized/reductive state that may hinder NFκB binding activity decreasing NFκB association with TNF-α promoter sequence.

The most intriguing aspect of our work was the ability of GSH-C4 to block inflammatory response even in cells that do not belong to the immune system, such as adipocytes and myotubes. In fact, it is known that inflammation is not limited to immune cells and can involve also adipose, and skeletal muscle tissues (75). Systemic inflammation is observed, accompanied by production of pro-inflammatory cytokines that can inhibit adipocyte differentiation (23, 76). Moreover, we have previously reported a significant production of pro-inflammatory cytokines (IL-6 and TNF-α) in visceral adipose tissue of 24 month-old mice and in 21 day-old adipocytes compared with 1 month-old mice and 8 day-old adipocytes, respectively (25). Similarly, increasing evidence supports that inflammation can also occur in skeletal muscle tissue during aging or in obesity, potentially driving immune cell infiltration, pro-inflammatory cytokine production, insulin resistance, and inability to complete the myogenesis process (77, 78). Indeed, we previously demonstrated an increase of IL-6 and TNF-α production in skeletal muscle of old mice paralleled by increased hallmarks of oxidative damage (28). In line with this evidence, we found that the treatment with GSH-C4, by altering the intracellular redox state, counteracted the activation of p-NFκB (p65), its nuclear translocation, and consequently the transcription of the inflammatory cytokine genes, such as TNF-α in both experimental systems used. Moreover, we previously published that the GSH/GSSG ratio decreases, shifting the redox balance toward oxidizing conditions, both during adipogenesis of 3T3-L1 cells (30) and in aged muscle tissue (28). These effects are of particular importance due to the systemic/detrimental role of inflammation in obesity and related diseases and in skeletal muscle degeneration. Moreover, for adipocytes we demonstrated a remarkable inhibition of the chemokine MCP-1 that is produced by white fat depot and functions as a potent chemotactic factor for monocytes (51) infiltrating the adipose tissue of obese mice (79), identified as molecular factor concurring to insulin resistance (52).

Overall our findings support an underlying role of GSH in the inflammatory response of both immune and non-immune cells, via the pivotal role of NFkB. Further, we propose that GSH-C4 may work as a promising candidate drug (particularly used in low doses) to prevent or treat the onset and progression of inflammatory diseases (i.e., cancer, aging, cystic fibrosis, cardiovascular, and neurodegenerative diseases) where oxidative/nitrosative stress and alterations of GSH balance have a predominant role (80).

## Author Contributions

MC and AP wrote the manuscript. DL and SB design, performed experiments, collected the data, and performed the analysis. AF and MEM read and revised the manuscript. PC, MM, and GD analyzed the data.

### Conflict of Interest Statement

The authors declare that the research was conducted in the absence of any commercial or financial relationships that could be construed as a potential conflict of interest.

## Abbreviations

GSH: Glutathione
LPS: lipopolisaccharide
NAC: N-acetyl-L-cysteine
GSH-C4: N-butanoyl glutathione
NFκB: nuclear factor kappa β transcription factor
ROS/RNS: reactive oxygen and nitrogen species
TNF-α: tumor necrosis factor
vAT: visceral adipose tissue
γ-GCS: γ-glutamyl-l-cysteinyl-ethyl ester.
