# Corrigendum: GSH-C4 Acts as Anti-inflammatory Drug in Different Models of Canonical and Cell Autonomous Inflammation Through NFκB Inhibition

**DOI:** 10.3389/fimmu.2019.01481

**Published:** 2019-07-02

**Authors:** Dolores Limongi, Sara Baldelli, Paola Checconi, Maria Elena Marcocci, Giovanna De Chiara, Alessandra Fraternale, Mauro Magnani, Maria Rosa Ciriolo, Anna Teresa Palamara

**Affiliations:** ^1^Department of Human Sciences and Promotion of the Quality of Life, IRCCS San Raffaele Pisana, San Raffaele Roma Open University, Rome, Italy; ^2^Department of Public Health and Infectious Diseases, Sapienza University of Rome, Rome, Italy; ^3^Institute of Translational Pharmacology, National Research Council Rome, Rome, Italy; ^4^University of Urbino Carlo Bo, Department of Biomolecular Sciences, Urbino, Italy; ^5^Department of Biology, University of Rome “Tor Vergata”, Rome, Italy; ^6^IRCCS San Raffaele Pisana, Rome, Italy; ^7^Institute Pasteur-Fondazione Cenci Bolognetti, Rome, Italy

**Keywords:** glutathione, macrophage, adipocytes, myocytes, cytokine

In the original article, there was a mistake in Supplementary Figure 1 and Supplementary Figure 2 as published. In the Supplementary Figure 1 the yellow sub-lines were still present. In the Supplementary Figure 2 the images were wrong. The corrected Supplementary Figures 1 and 2 appear below.

**Figure:**
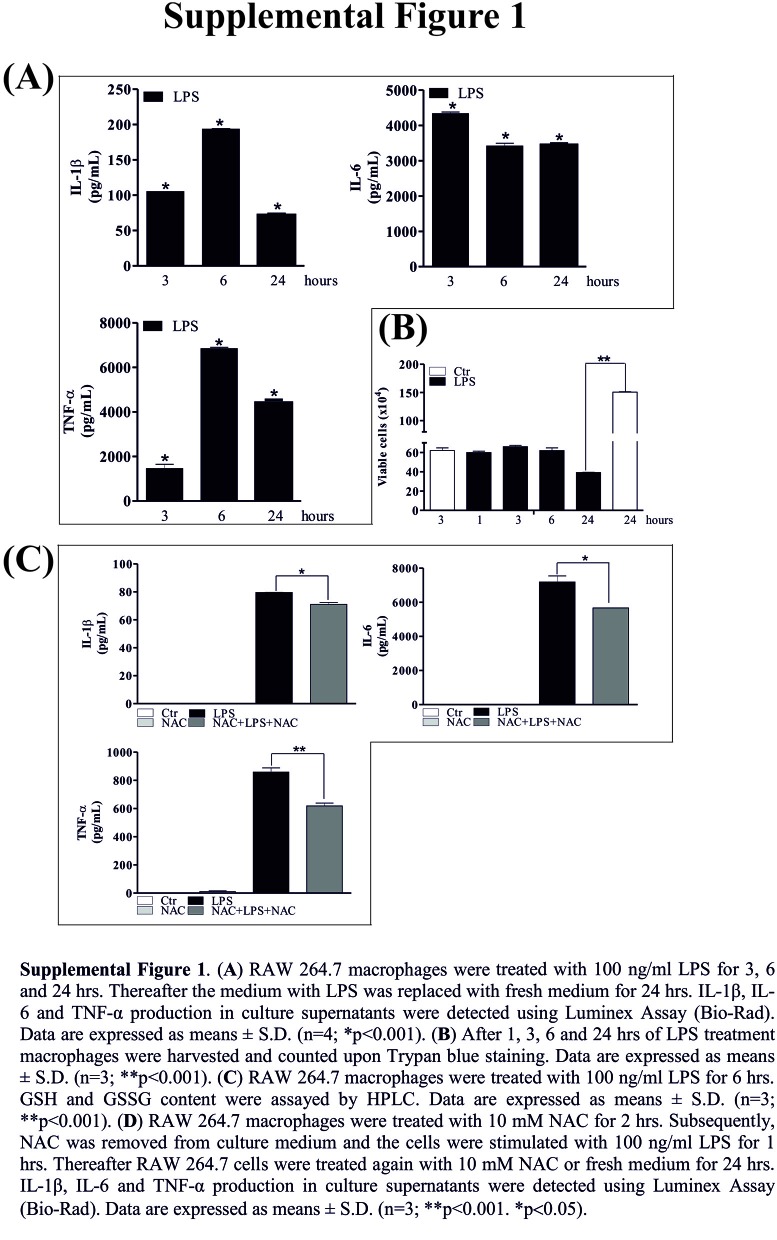


**Figure:**
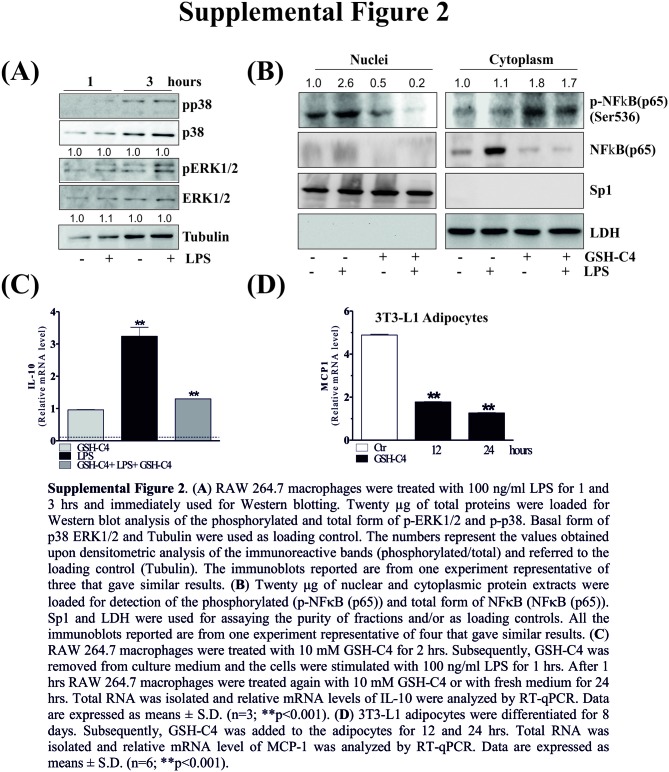


Additionally, an author name was incorrectly spelled as “Mariaelena Marcocci.” The correct spelling is “Maria Elena Marcocci.”

There was also an error regarding the affiliations for Anna Teresa Palamara. As well as having affiliations 6 and 7, they should also have affiliation 2.

The authors apologize for these errors and state that they do not change the scientific conclusions of the article in any way. The original article has been updated.

