# Supplementary figures and images for: GSH-C4 Acts as Anti-inflammatory Drug in Different Models of Canonical and Cell Autonomous Inflammation Through NFκB Inhibition
Source: Front Immunol. 2019 Feb 6;10:155. doi: 10.3389/fimmu.2019.00155 (PMC6372722; doi:10.3389/fimmu.2019.00155)

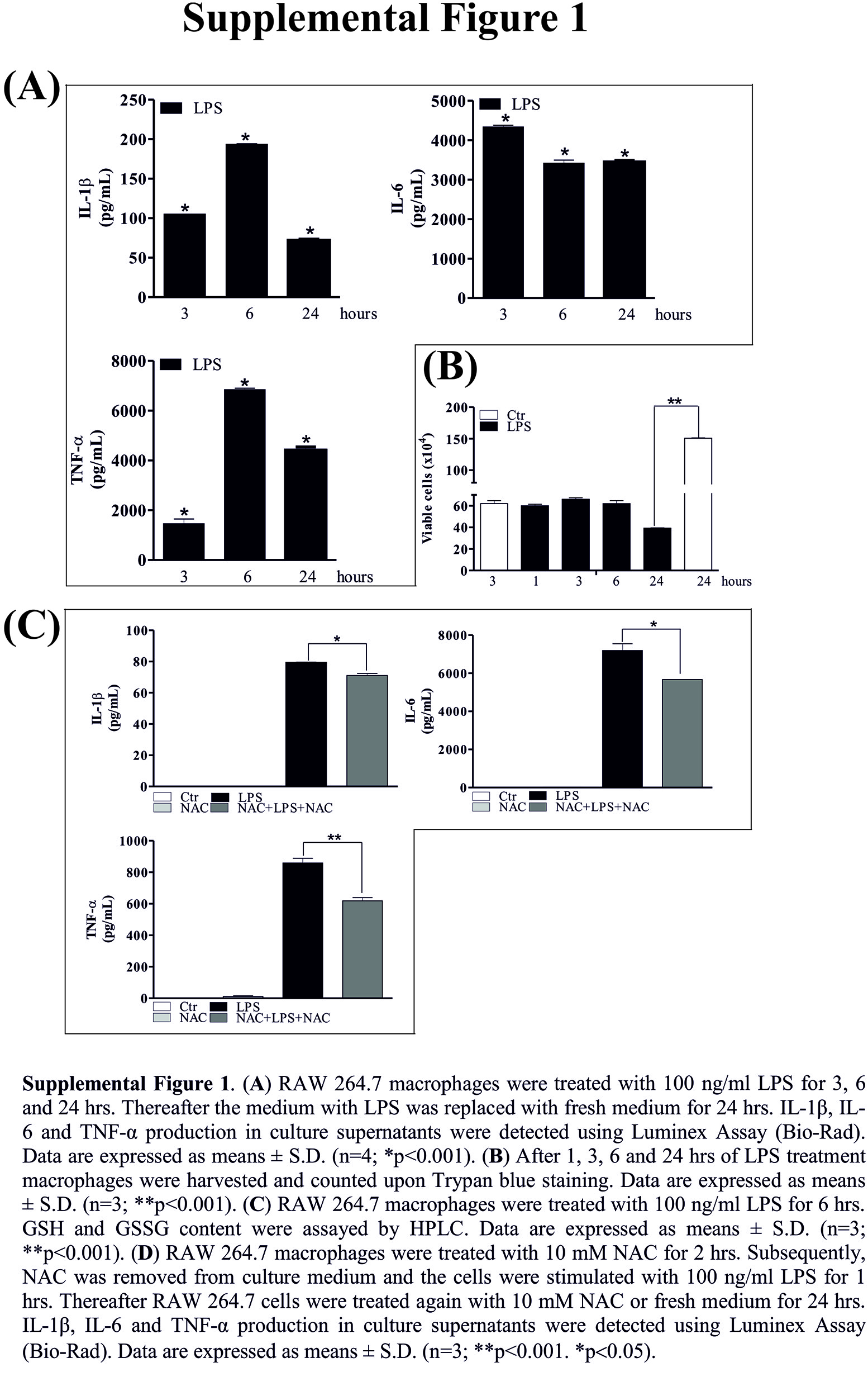

Supplement: Supplementary file 1 [file Image_1.tif]

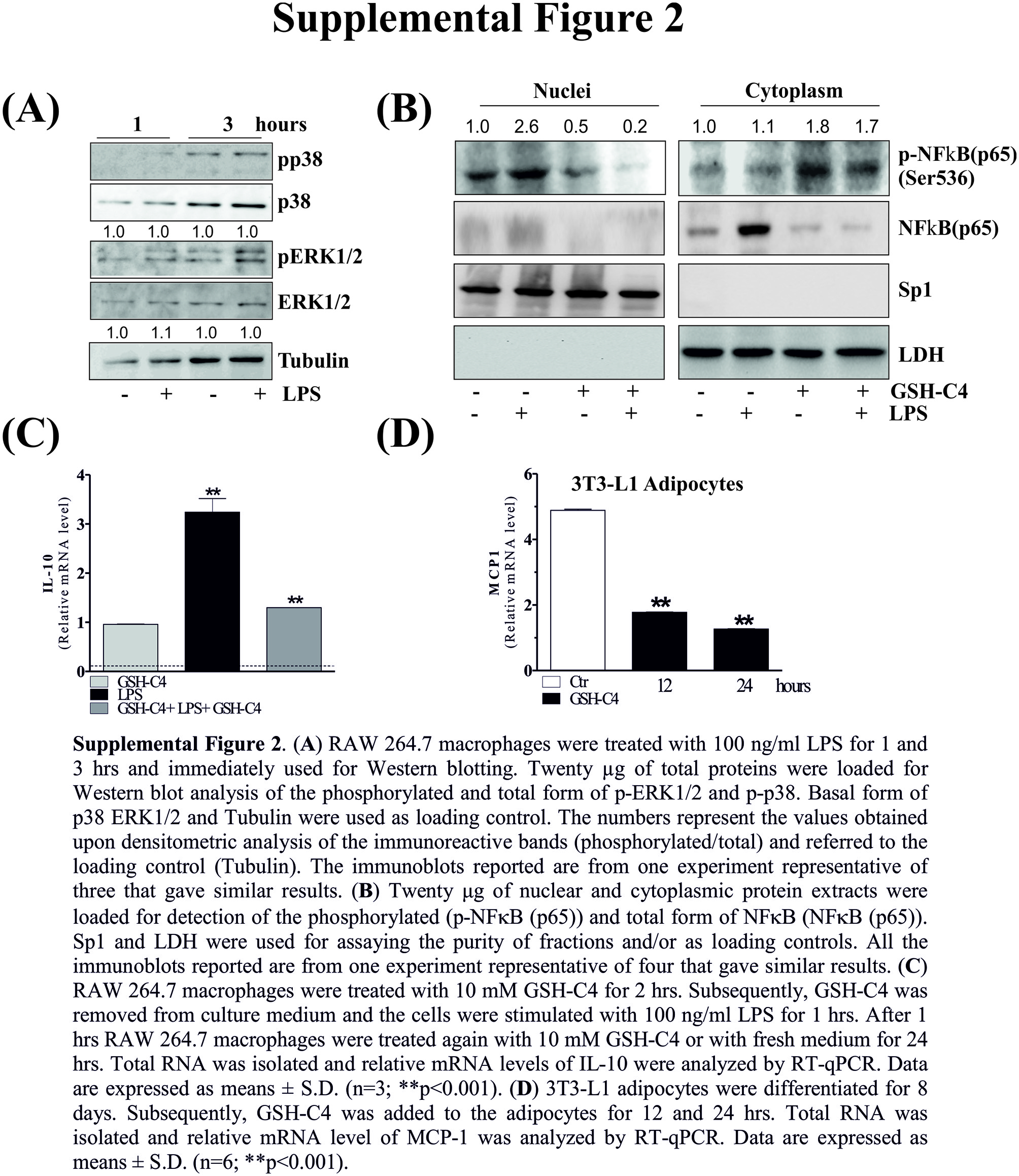

Supplement: Supplementary file 2 [file Image_2.tif]
